# Is the selfish life-cycle model more applicable in Japan and, if so, why? A literature survey

**DOI:** 10.1007/s11150-020-09511-0

**Published:** 2020-10-20

**Authors:** Charles Yuji Horioka

**Affiliations:** 1 Research Institute for Economics and Business Administration, Kobe University, 2-1, Rokkodai-cho, Nada-ku, Kobe, Hyogo, 657-8501 Japan; 2 Asian Growth Research Institute, Kitakyushu, Japan; 3 Institute of Social and Economic Research, Osaka University, Ibaraki, Japan; 4 National Bureau of Economic Research, Cambridge, MA USA

**Keywords:** Altruism, Bequest motives, Household saving, Intergenerational transfers, Japan, Life-cycle model, D11, D12, D14, D15, D64, E21, J14

## Abstract

The selfish life-cycle model or hypothesis is, together with the dynasty or altruism model, the most widely used theoretical model of household behavior in economics, but does this model apply in the case of a country like Japan, which is said to have closer family ties than other countries? In this paper, we first provide a brief exposition of the simplest version of the selfish life-cycle model and then survey the literature on household saving and bequest behavior in Japan in order to answer this question. The paper finds that almost all of the available evidence suggests that the selfish life-cycle model applies to at least some extent in all countries but that there is more consistent support for this model in Japan than in the United States and other countries. It then explores possible explanations for why the life-cycle model is more consistently supported in Japan than in other countries, attributing this finding to government policies, institutional factors, economic factors, demographic factors, and cultural factors. Finally, it shows that the findings of the paper have many important implications for economic modeling and for government tax and expenditure policies.

## Introduction

The selfish life-cycle model or hypothesis is, together with the dynasty or altruism model of Barro (1974) that assumes the presence of intergenerational altruism, the most widely used theoretical model of household behavior in economics. Many researchers have investigated whether or not this model applies in North America, Europe, and elsewhere (see, for example, Modigliani (1975), Deaton (1992, 2005), Browning and Lusardi (1996), Hayashi (1997b), Attanasio (1999), Browning and Crossley (2001), Baranzini (2005), Attanasio and Weber (2010), and Jappelli and Pistaferri (2017) for useful surveys). However, whether or not the selfish life-cycle model applies in the case of a country like Japan, which is said to have closer family ties than other countries, is another question.

The purpose of this paper is to provide a brief exposition of the simplest version of the selfish life-cycle model and to then survey the literature on household saving and bequest behavior in Japan to shed light on whether or not the selfish life-cycle model applies in the case of Japan. There have been many comprehensive surveys of the literature on household saving, consumption, and bequest behavior in Japan (for example, Hayashi (1986, 1992, 1997a), Horioka (1984, 1990, 1993, 2008a), and Ogawa and Horioka (1996)), but this paper is unique in focusing on the question of whether or not the selfish life-cycle model applies and in surveying the literature on a wide variety of approaches including not only studies of saving behavior (e.g., studies of the impact of the age structure of the population on the saving rate, the saving behavior of the retired elderly, saving motives, and the importance of borrowing (liquidity) constraints) but also studies of bequest behavior (e.g., studies of the prevalence of bequests, bequest motives, and tests of altruism). I emphasize my own research because, over the years, I have tried a variety of approaches to test the validity of the selfish life-cycle model.

One view that has its adherents even today is that the laws of Western economics (including the selfish life-cycle model) do not apply in the case of Japan, that the Japanese are not rational utility maximizers, and that the economic behavior of the Japanese is largely determined by the country’s unique culture and social norms, especially its Confucian heritage (see Morishima 1982, and Katzner 1999, 2008).^1^ For example, a common view is that Japan’s high household saving rate is attributable to national character, culture, or Confucian teachings concerning frugality (see, for example, Horioka 1990, 2019; Garon 1997, pp. 164–165). Thus, it is of great interest to examine whether or not the selfish life-cycle model applies in the case of a non-Western country such as Japan despite enormous differences in culture and social norms.

To preview the main findings of this paper, it finds that almost all of the available evidence suggests that the selfish life-cycle model applies at least to some extent in all countries but that there is more consistent support for this model in Japan than in the United Sates and other countries. Thus, the answer to the question posed in the title of this paper is an unqualified “yes.” The paper then explores possible explanations for why the selfish life-cycle model is more consistently supported in Japan than in other countries, attributing this finding to government policies, institutional factors, economic factors, demographic factors, and cultural factors. Finally, it shows that the findings of the paper have many important implications for economic modeling and for government tax and expenditure policies.

This paper is organized as follows: we provide a brief exposition of the simplest version of the selfish life-cycle model in Section 2; we survey the literature on the impact of the age structure of the population on the saving rate in Section 3, that on the saving behavior of the retired elderly in Section 4, that on saving motives in Section 5, that on the prevalence of bequests in Section 6, that on bequest motives in Section 7, that on tests of altruism in Section 8, and that on the importance of borrowing constraints in Section 9; we explore possible explanations for why there is more consistent support for the selfish life-cycle model in Japan than there is in other countries in Sections 10–16; and we summarize our findings and discuss the implications thereof for economic modelling and for government tax and expenditure policies in Section 17.

## The selfish life-cycle model

In this section, we provide a brief exposition of the simplest version of the selfish life-cycle model, which is primarily attributable to Franco Modigliani and his collaborators (for more details, see Modigliani and Brumberg (1954, 1980), Ando and Modigliani (1963), Modigliani (1966), Modigliani (1975), Baranzini (2005), and Deaton (2005)).

In a nutshell, this model assumes that individuals are rational, forward-looking, and selfish, that they maximize their own lifetime utility subject to a lifetime budget constraint, and that they neither receive nor leave bequests and other intergenerational transfers. Since most individuals retire at some point in their lives, this model requires individuals to save part of their earnings during their working years to prepare for living expenses during retirement and to finance their living expenses during retirement by drawing down (decumulating) their previously accumulated assets.

**Fig. 1:**
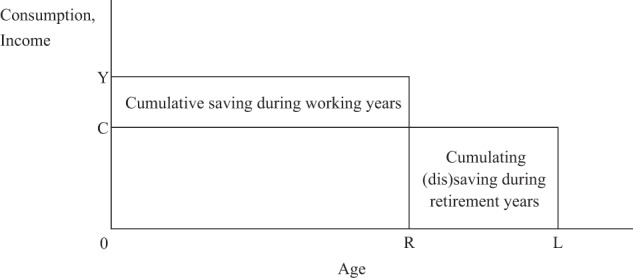
The selfish life-cycle model

In order to simplify the model as much as possible, we ignore the childhood years and assume that individuals graduate from school and start working at age 0 and that they work from age 0 until age R, earning Y yen each year, part of which they use to finance their current consumption and part of which they save in preparation for their living expenses during retirement. We further assume that individuals retire at the exogenous retirement age R, that they finance their living expenses during retirement by drawing down their previously accumulated assets, and that they die with certainty at age L. Finally, we assume that individuals smooth their consumption throughout their lives, consuming C yen per year from age 0 until age L, and that they are selfish, receiving no bequests or inter vivos transfers from their parents and leaving no bequests or inter vivos transfers to their children (see Fig. 1).

If we assume that the interest rate is zero for the sake of simplicity, these assumptions imply that lifetime income will be Y × R and that lifetime consumption will be C × L. Thus, the lifetime budget constraint can be expressed as follows:

1 $${\mathrm{Y}} \times {\mathrm{R}} = {\mathrm{C}} \times {\mathrm{L}}.$$

If Eq. (1) is solved for C, we can obtain the following expression for C, which denotes the annual amount of consumption:

2 $${\mathrm{C}} =\left({{\mathrm{R}}/{\mathrm{L}}} \right) \times {\mathrm{Y}}.$$

In words, consumption will be a constant fraction of income, with that fraction being equal to the ratio of one’s working years to total lifespan.

SW, the annual saving of working individuals can be calculated by subtracting consumption C from income Y and substituting the expression in Eq. (2) for C:

3 $${\mathrm{SW}} = {\mathrm{Y}}-{\mathrm{C}} = \left( {1-{\mathrm{R}}/{\mathrm{L}}} \right) \times {\mathrm{Y}} = \left( {\left( {{\mathrm{L}}-{\mathrm{R}}} \right)/{\mathrm{L}}} \right) \times {\mathrm{Y}}.$$

In words, the saving of working individuals will be a constant fraction of their income, with that fraction being equal to the ratio of their retirement span to total lifespan.

Moreover, since individuals are assumed to work for R years, CSW, the cumulative saving of working individuals, is as follows:

4 $${\mathrm{CSW}} = {\mathrm{R}} \times \left( {\left( {{\mathrm{L}}-{\mathrm{R}}} \right)/{\mathrm{L}}} \right) \times {\mathrm{Y}} = \left( {{\mathrm{L}}-{\mathrm{R}}} \right) \times \left( {{\mathrm{R/L}}} \right) \times {\mathrm{Y}}.$$

As for retired individuals, since they have no income but need to consume, their consumption will be financed entirely by drawing down their previously accumulated saving. Thus, SR, the annual (dis)saving of retired individuals, is as follows:

5 $${\mathrm{SR}} = -{\mathrm{C}} = -\left( {{\mathrm{R}}/{\mathrm{L}}} \right) \times {\mathrm{Y}}.$$

Moreover, since the retirement span of retired individuals is (L – R) years, CSR, the cumulative (dis)saving of retired individuals, is as follows:

6 $${\mathrm{CSR}} = -\left( {{\mathrm{L}}-{\mathrm{R}}} \right) \times \left( {{\mathrm{R}}/{\mathrm{L}}} \right) \times {\mathrm{Y}}.$$

As can be seen by comparing Eqs. (4) and (6), the cumulative saving of working individuals and the cumulative (dis)saving of retired individuals are precisely equal to one another in absolute value but have opposite signs. This confirms that the lifetime budget constraint of individuals is satisfied. This is the same as saying that the area of the rectangle showing the cumulative saving of individuals during their working years is exactly equal to the area of the rectangle showing the cumulative (dis)saving of individuals during their retirement years in Fig. 1.

Next, we wish to derive the saving amount and saving rate of the household sector as a whole on the assumption that all households are identical. From Eqs (3) and (5), AS, the aggregate saving of the household sector as a whole, is as follows:

7 $${\mathrm{AS}} = {\mathrm{POP}}\left( {0,\,{\mathrm{R}}} \right) \times \left( {\left( {{\mathrm{L}}-{\mathrm{R}}} \right)/{\mathrm{L}}} \right) \times {\mathrm{Y}}-{\mathrm{POP}}\left( {{\mathrm{R}},\,{\mathrm{L}}} \right) \times \left( {{\mathrm{R}}/{\mathrm{L}}} \right) \times {\mathrm{Y}}.$$

where POP(0, R) = the population aged 0 to R (the working-age population); POP(R, L) = the population aged R to L (the retirement-age population).

Furthermore, AY, the aggregate income of the household sector as a whole, is as follows:

8 $${\mathrm{AY}} = {\mathrm{POP}}\left( {0,\,{\mathrm{R}}} \right) \times {\mathrm{Y}}.$$

Thus, ASY, the saving rate of the household sector as a whole, is as follows:

9 $${\mathrm{ASY}} = {\mathrm{AS}}/{\mathrm{AY}} = \left( {{\mathrm{L}}-{\mathrm{R}}} \right)/{\mathrm{L}}-\left[ {{\mathrm{POP}}\left( {{\mathrm{R}},\,{\mathrm{L}}} \right)/{\mathrm{POP}}\left( {0,\,{\mathrm{R}}} \right)} \right] \times \left( {{\mathrm{R}}/{\mathrm{L}}} \right).$$

In other words, the saving rate of the household sector as a whole should be a function of the ratio of the retirement-age population to the working-age population, and the higher is this ratio, the lower should be the household saving rate. Moreover, the derivative of the household saving rate with respect to this ratio should equal the negative of the ratio of one’s working years to total lifespan.

Thus, we can test whether the selfish life-cycle model applies in the real world by examining whether or not the age structure of the population (in particular, the ratio of the retirement-age population to the working-age population) has the expected impact on the household saving rate.

Thus far, we have simplified our theoretical model by making the following assumptions:

1. There is no economic growth.
2. There is no public old-age pension system.
3. There is no lifespan uncertainty.

The age structure of the population will have an impact on the household saving rate even if these simplifying assumptions are relaxed, but what will change is that now other factors will also affect the household saving rate.

1. If the economy is growing, cohorts born later will have higher lifetime incomes than cohorts born earlier, and thus the aggregate saving of cohorts that are working and saving will exceed the aggregate dissaving of cohorts that are retired and dissaving in absolute value. As a result, if the economy is growing, the aggregate saving of the household sector as a whole will be positive, and the higher is the economic growth rate, the greater will be the aggregate saving of the household sector as a whole.
2. If a public old-age pension system is introduced, there will be less need to save in preparation for living expenses during retirement, and thus one would expect the saving of working-age individuals and the (dis)saving of retired individuals to be less than in a world with no public old-age pension system (see, for example, Feldstein (1974)).
3. If lifespan is uncertain and individuals are risk-averse, one would expect the saving of working-age individuals to be greater and the dissaving of retirement-age individuals to be less than in a world with no lifespan uncertainty because individuals will be afraid of running out of money before they die (see Davies (1981)). This will lead individuals to leave unintended or accidental bequests unless they are able to completely annuitize their wealth.

## Evidence on the impact of the age structure of the population on the saving rate

As we showed in the previous section, if the selfish life-cycle model applies, the household saving rate should be a decreasing function of the ratio of the retirement-age population to the working-age population. Thus, we can shed light on the applicability of the selfish life-cycle model by investigating whether or not the age structure of the population has the expected impact on the saving rate.

The author has used various types of data to investigate the impact of the age structure of the population on the saving rate, and in this section, we survey this body of work and consider whether or not the findings are consistent with the selfish life-cycle model. In subsection 3.1, we discuss papers that make use of cross-country or cross-provincial data, while in subsection 3.2, we discuss papers that make use of time-series data for Japan.

### Evidence based on cross-country and cross-provincial data

Horioka (1986, 1989) uses data on the member countries of the Organisation for Economic Co-operation and Development (OECD) for the 1975–84 period from the OECD to analyze the determinants of the private saving rate and finds that the ratio of the retirement-age population to the working-age population has a negative and statistically significant impact on the private saving rate.

Based on his estimation results, Horioka (1986, 1989) calculates the contribution of each factor to the difference between Japan’s private saving rate and the OECD-wide average and to the U.S.-Japan saving rate gap (see Table 1). During the 1975–84 time period, Japan’s population was the youngest and her retirement-age population to working-age population ratio was the lowest among all OECD member countries at the time, and as Table 1 shows, this can explain virtually all of the difference between Japan’s private saving rate and the OECD-wide average and about half of the U.S.-Japan saving rate gap.

**Table 1:** A Decomposition of the private saving rate gap between Japan and other countries

Factor	U.S.–Japan gap	Gap between Japan and OECD-wide average
The contribution of a high income growth rate	+0.45	+0.47
The contribution of a low retirement-age population ratio	+5.45	+6.90
The contribution of a low dependent-age population ratio	+1.64	+1.07
The contribution of a high labor force participation rate of the elderly	−3.94	−3.89
The contribution of a low per capita income level	+4.36	+1.29
Subtotal	+7.96	+5.85
Unexplained residual	+2.90	+0.95
Total private saving rate gap	+10.86	+6.80

The figures denote the contribution of each factor to the private saving rate gap (in percentage points)

Source: Horioka (1986, 1989)

Similarly, Horioka and Terada-Hagiwara (2012) use panel data on twelve Asian countries for the 1966–2007 period from Penn World Tables to analyze the determinants of the domestic saving rate, and as in the case of Horioka (1986, 1989), they find that the ratio of the retirement-age population to the working-age population has a negative and statistically significant impact on the domestic saving rate.

Finally, Horioka and Wan (2007) use provincial panel data for the 1995–2004 period from a household survey to analyze the determinants of the household saving rate in China and find that the impact of the ratio of the retirement-age population to the working-age population is negative, as expected, but often not statistically significant.

The reader is referred to Loayza et al. (2000) for a survey of more recent research and for a comprehensive econometric analysis. Virtually all of the papers surveyed in this paper as well as the analysis in this paper itself find, as expected, that the ratio of the retirement-age population to the working-age population has a negative and statistically significant impact on the private saving rate.

### Evidence based on time-series data

Horioka (1997) uses time-series data for the 1955–93 period from Japan’s National Accounts to analyze the determinants of Japan’s household saving rate and finds that there is a cointegrating relationship between the household saving rate and the ratio of the retirement-age population to the working-age population and that this ratio has a negative and statistically significant impact on the household saving rate (see Horioka (1991) and Koga (2006) for similar studies for Japan and Modigliani and Cao (2004) for a similar study for China, all of which obtain broadly consistent results).

**Fig. 2:**
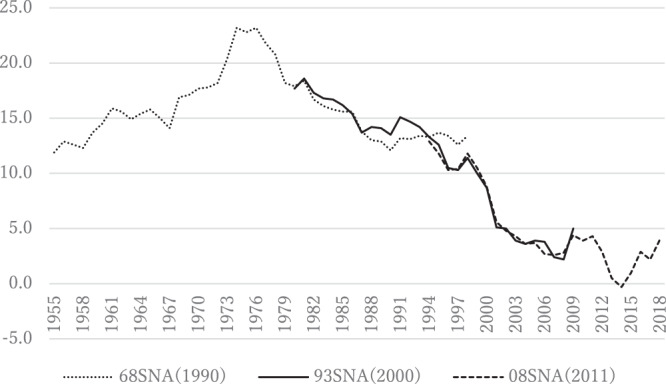
Trends over time in Japan’s household saving rate, 1955–2018. Note: This figure shows trends over time in the household saving rate, which is defined as household saving as a share of household disposable income (in percent). SNA denotes System of National Accounts. Source: Economic and Social Research Institute, Cabinet Office, Government of Japan, “Annual Report on National Accounts,” various issues

Figure 2 shows trends over time in National Accounts data on Japan’s household saving rate for the 1955–2018 period, and as can be seen from this figure, Japan’s household saving rate showed a steady upward trend from 1955 until the mid-1970s, peaking at 23.2%, and showed a steady downward trend thereafter, sometimes even becoming negative.

The ratio of the retirement-age population to the working-age population in Japan has shown a steady upward trend over time so Horioka’s (1997) finding that this ratio has a negative and statistically significant impact on the household saving rate implies that the upward trend in this ratio can explain the downward trend in Japan’s household saving rate since the mid-1970s and that further increases in this ratio will cause further declines in Japan’s household saving rate in future years (see Horioka 1989, 1992).

As for why Japan’s household saving rate showed a steady upward trend from 1955 until the mid-1970s, Horioka (2008a) argues that it was due to the steady downward trend in the ratio of minors (those aged 0 to 19) to the working-age population during the same period, which in turn was due to the decline in the fertility rate. The ratio of minors to the working-age population would be expected to have a negative impact on the household saving rate, as in the case of the ratio of the retirement-age population to the working-age population, because minors contribute to consumption without contributing to income.

### Summary

In this section, we showed that the ratio of the retirement-age population to the working-age population has a negative and statistically significant impact on the saving rate whether one uses cross-country data or time-series data. This not only suggests that the selfish life-cycle model applies in the case of Japan but also that the low ratio of the retirement-age population to the working-age population can explain why Japan’s household saving rate was so high in the past and that the sharp increase in this ratio can explain the sharp decline in Japan’s household saving rate since the mid-1970s. Thus, the selfish life-cycle model can explain the level of, as well as trends over time in, Japan’s household saving rate. However, we should note that the selfish life-cycle model is not the only theoretical model that predicts that the age structure of the population will have a significant impact on the household saving rate and thus that, although we can conclude that the evidence is consistent with the selfish life-cycle model, we cannot assert that we have unequivocably established that the selfish life-cycle model applies in the case of Japan.

## Evidence on the saving behavior of the retired elderly

The selfish life-cycle model assumes that working individuals save and that retired individuals dissave. Thus, we can shed light on the applicability of the selfish life-cycle model by examining the saving behavior of the retired elderly (see Weil (1994) and De Nardi et al. (2016) for excellent surveys of this literature). In this section, we survey studies that examine the saving behavior of the retired elderly in Japan and consider whether or not their findings are consistent with the selfish life-cycle model.

All of the papers we surveyed find that the retired elderly in Japan dissave, at least after a certain age, which constitutes evidence in favor of the selfish life-cycle model. For example, Hayashi et al. (1988) carefully analyze household-level data from the “National Survey of Family Income and Expenditure,” which is conducted every five years by the Statistics Bureau of the Japanese Ministry of Internal Affairs and Communications and find that the elderly start dissaving after the age of 80 whether they live in nuclear households or extended (three-generation) households. However, Hayashi et al. (1988) do not take account of the employment status of respondents, and if they had confined their sample to the retired elderly, they are likely to have found that the Japanese elderly start dissaving at an earlier age.

Horioka (2006, 2010) and Horioka and Niimi (2017) analyze tabulated data on the retired elderly from the “Family Income and Expenditure Survey,” which is conducted by the Statistic Bureau of the Japanese Ministry of Internal Affairs and Communications, and find that the saving rate of such households has fluctuated in the −40 to −10% range and that their decumulation rate of financial assets has fluctuated in the 1 to 3% range since 2000.^2^ These findings constitute convincing evidence that the retired elderly in Japan are dissaving and suggest that the selfish life-cycle model applies in the case of Japan (see also Horioka et al. (1996), Horioka and Niimi (2017), and Niimi and Horioka (2019)).

However, we should note that the selfish life-cycle model is not the only theoretical model that predicts that the retired elderly should dissave and thus that, although we can conclude that the evidence is consistent with the selfish life-cycle model, we cannot assert that we have unequivocably established that the selfish life-cycle model applies in the case of Japan.

## Evidence on saving motives

The simplest version of the selfish life-cycle model implies that individuals should be saving primarily for living expenses during retirement and that they should not be saving to leave bequests to their children. Thus, we can shed light on the applicability of the selfish life-cycle model by looking at the relative importance of saving for individual motives. In this section, we survey studies that attempt to estimate the contribution of saving for each motive to total household saving in Japan and other countries and consider whether or not the findings are consistent with the selfish life-cycle model. Our findings support the selfish life-cycle model because they show that saving for life-cycle motives such as retirement are much more important than saving for bequests in Japan as well as in many other countries.

Before turning to our findings, however, let us first explain the methodology we used to calculate the amount of saving for each motive. If individuals cannot realize a given motive with only their current income, they need to rely on saving. Moreover, at any given time, there will be individuals who are saving in order to prepare for a given motive as well as individuals who are dissaving to realize the same motive. For example, at any given time, there will be pre-retirement individuals who are saving for retirement as well as post-retirement individuals who are dissaving for retirement. Thus, the contribution that saving for a given motive makes to aggregate household saving is net saving for that motive, which can be calculated as gross saving for that motive minus dissaving for that motive. Mathematically,

10 $${\mathrm{NS}} = {\mathrm{net}}\,{\mathrm{saving}}\,{\mathrm{for}}\,{\mathrm{a}}\,{\mathrm{given}}\,{\mathrm{motive}} = {\mathrm{GS}}-{\mathrm{DS}},$$

where GS = gross saving for a given motive; DS = dissaving for a given motive.

Furthermore, there are two ways in which one can use saving to help realize a given motive. The first way is to rely on one’s own assets, and in the case of this way, one accumulates the financial assets needed to realize the motive in question beforehand and draws down those assets in order to realize that motive. The other way is to rely on borrowing, and in this case, one borrows the funds needed to realize the motive in question, uses those funds to realize that motive, and repays the loan little by little after realizing the motive (note that loan repayments (repayment of the principal only) are a form of saving). What should be noted is that the saving is done before the realization of the motive when one relies on one’s own wealth and that it is done after the realization of the motive when one relies on borrowing.

The gross saving and dissaving for a given motive in the case of the two financing methods are shown in Table 2, and as can be seen from this table, the gross saving for a given motive equals the sum of saving in the form of the accumulation of financial assets and saving in the form of loan repayments. Similarly, dissaving for a given motive equals dissaving in the form of the decumulation of financial assets and dissaving in the form of new borrowings.^3^ Moreover, as noted earlier, net saving for a given motive equals gross saving for that motive minus dissaving for that motive.

**Table 2:** Saving for specific motives

Financing method	Gross saving	Dissaving
Own wealth	Accumulation of financial assets	Decumulation of financial assets
Borrowing	Loan repayments	New borrowing

The former Institute of Posts and Telecommunications Policy of the former Ministry of Posts and Telecommunications of the Japanese Government conducted a number of surveys of household saving behavior including the “Survey of the Financial Asset Choice of Households,” which was conducted in Japan every two years, and the “U.S.–Japan Comparison Survey of Saving,” which was conducted simultaneously in the United States and Japan in 1996. Both of these surveys are unique in asking respondents to provide information on the amount of saving, dissaving, new borrowings, and loan repayments for each motive. Horioka and Watanabe (1997, 1998) and Horioka et al. (1998, 2000) use the methodology described above in conjunction with data from the 1994 “Survey of the Financial Asset Choice of Households” and the 1996 “U.S.-Japan Comparison Survey of Saving,” respectively, to calculate the contribution of saving for each motive to aggregate household saving (see Horioka (1985) for an analysis of saving for one’s children’s educational expenses, Horioka (1987) and Grossbard (2015) for an analysis of saving for one’s children’s marriage expenses, Horioka (1988) for an analysis of saving for housing purchase, and Horioka and Okui (1999) for an analysis of saving for retirement).^4^

Table 3 shows data from Horioka and Watanabe (1997, 1998) and Horioka et al. (1998, 2000) on the contribution of net saving for each motive to total household saving from the two aforementioned surveys. If the selfish life-cycle model applies, individuals should be saving primarily for the retirement motive, and as this table shows, net saving for the retirement motive accounts for a full 62.23–62.50 and 30.84% of total household saving in Japan and the United States, respectively, and that it is by far the dominant component of household saving in both countries. Thus, the selfish life-cycle model seems to apply in both countries. However, the share of retirement-related saving in Japan is more than twice what it is in the United States, which suggests that the selfish life-cycle model applies to a much greater extent in Japan than it does in the United States.^5^

**Table 3:** A U.S.–Japan comparison of the share of net saving for each motive

Saving motive	Japan (1994)	Japan (1996)	U.S. (1996)
Retirement	62.50	62.23	30.84
Precautionary	55.99	41.18	27.93
Children’s education	8.93	8.77	−0.14
Children’s marriage	7.52	7.31	2.87
Housing purchase	−20.21	−15.57	14.60
Consumer durable purchases	−3.75	1.54	4.20
Leisure	−0.44	2.44	6.35
Payment of taxes	−1.54	0.25	6.40
Independent business	0.10	−0.37	2.59
Bequests	3.23	1.50	5.04
Other	−12.33	−9.29	−0.66
Total	100.00	100.00	100.00

The figures denote the share of net saving for each motive in total household saving (in percent). The figure for “Precautionary” denotes the sum of the figures for “Illness” and “Peace of mind”.

Sources: Horioka (1998, 1999) and Horioka et al. (1998, 2000)

If the simplest version of the selfish life-cycle model applies, individuals should not leave a bequest to their children and should therefore not be saving in order to leave a bequest to them. As can be seen from Table 3, the share of net saving for the bequest motive is 1.50–3.23% in Japan and 5.04% in the United States, and thus its share is low in both countries but especially in Japan. Thus, our findings concerning saving for the bequest motive also suggest that the selfish life-cycle model applies in both Japan and the United States but that it is especially applicable in the case of Japan.^6^

A closely related paper is Gourinchas and Parker (2002), which analyzes how the proportions of precautionary saving (buffer saving) and retirement saving (life-cycle saving) evolve over the life cycle using data for the United States and finds that precautionary saving decreases sharply with age whereas retirement saving increases sharply with age and that precautionary saving comprises the lion’s share of the target level of liquid wealth until about the age of 40.

Another closely related paper is Schunk (2009), which uses micro data from the SAVE data set to analyze motives for saving in Germany. Schunk (2009) finds that the most important motive for saving in Germany is the precautionary motive, with 62% of respondents feeling that this motive is “very important,” followed by the old-age provision motive (59%), the motive to purchase a house (36%), and the bequest motive (20%). Horioka et al. (1998, 2000) presents roughly comparable data on the proportion of respondents saving for each motive, and they find that the most important motive is the retirement motive in both the United States and Japan, with 48.6 and 45.2% of respondents saving for this motive in the two countries, respectively, and that the bequest motive is far less important, with only 10.8 and 3.6% of respondents saving for this motive in the two countries, respectively. Thus, the retirement motive is much more important than the bequest motive in all three countries, but the ratio between the two is lowest in Germany (59 vs. 20%), intermediate in the United States (48.6 vs. 10.8%), and highest in Japan (45.2 vs. 3.6%).

Yet another closely related paper is Yao et al. (2011), which compares saving motives in China and the United States. Unfortunately, they do not consider saving for the bequest motive, but they find that saving for the retirement motive is more important for Chinese households than for American households in the lower income quantiles and that saving for the education motive is more important for Chinese households than for American households in all income quantiles. These results suggest that Chinese households are more similar to Japanese households than to American households in terms of the relative importance of saving for the retirement (and education) motives.

Yet another closely related paper about China is Chao et al. (2011), which finds that the life-cycle hypothesis can explain only 35% of the surge in Chinese household saving but that by adding to the model the strong motivation of young adults for buying a home and the financial support they receive from their parent for that purpose, their model can reproduce the high and increasing level of household saving since the mid-nineties.

Finally, Birkeland (2013) analyzes the saving motives of Dutch households and finds that saving for the precautionary motive is the most important motive for Dutch households, that saving for the retirement motive is the second most important motive, and that the inter vivos transfer motive and the bequest motive are less important.

To summarize, our findings concerning saving motives suggest that the selfish life-cycle model applies in all countries but that it applies to a greater extent in Japan (and perhaps also in China and the Netherlands) than it does in the United States and Germany.

## Evidence on the prevalence of bequests

The simplest version of the selfish life-cycle model assumes that individuals do not leave any bequests or other intergenerational transfers to their children so we can shed light on the applicability of the selfish life-cycle model by looking at the prevalence of bequests and other intergenerational transfers. In this section, we survey the literature on the prevalence of bequests and other intergenerational transfers in Japan and other countries and consider whether or not the findings are consistent with the selfish life-cycle model.

The most commonly used measure of the importance of bequests and other intergenerational transfers is the share of such transfers in total household wealth. This measure was first used by Kotlikoff and Summers (1981), and they obtained the shocking result that the share of intergenerational transfers in total household wealth amounts to a full 46 to 81 percent. Subsequently, many researchers have calculated this share using a variety of methodologies and data sources for a large number of countries. Davies and Shorrocks (2000) survey this literature and conclude that the majority of studies find that this share is 35 to 45% in the United States, roughly comparable to the United States in Canada, and somewhat higher in France.

Moreover, a number of researchers have tried to calculate the share of intergenerational transfers in total household wealth for the case of Japan. For example, Hayashi (1986) estimates this share to be at least 9%, Dekle (1989) estimates it to be 3–49%, Barthold and Ito (1992) estimate it to be at most 25–40%, Campbell (1997) estimates it to be 23–28%, and Horioka (2008b, 2008c, 2009) estimates it to be 15–18%. Thus, this share appears to be lower in Japan than it is in the United States and other countries, which suggests that bequests and other intergenerational transfers are less important quantitatively in Japan than they are in other countries. Thus, the findings concerning the quantitative importance of bequests also suggest that the selfish life-cycle model applies to a greater extent in Japan than it does in other countries.

Another approach for gauging the importance of bequests and other intergenerational transfers is to look not at the amounts of bequests actually left behind but to ask individuals about their bequest intentions. Osaka University has been conducting a household survey called the “Preference Parameters Study” in four countries (China, India, Japan, and the United States) since 2003, and fortunately, this survey contains several questions about bequests. The bequest data from this survey are analyzed in detail in Horioka (2014a, 2014b), and as can be seen from these papers, the proportion of respondents planning to leave a bequest to their children is by far the highest in India (87.05%), also relatively high in the United States (60.77%) and China (56.35%), and by far the lowest in Japan (31.44%). These findings reinforce our earlier conclusion that bequests are less prevalent in Japan than in other countries and that the selfish life-cycle model applies to a greater extent in Japan than it does in other countries. However, we should note that there is substantial heterogeneity in all countries and that, even in Japan, a substantial minority of households (nearly one-third) plan to leave bequests.

## Evidence on bequest motives

In the previous section, we presented our findings concerning the prevalence of bequests and other intergenerational transfers and concluded that they are less prevalent in Japan than in other countries. However, even if the selfish life-cycle model applies, individuals may still leave bequests and other intergenerational transfers to their children, and thus, even if we find that individuals do leave bequests and other intergenerational transfers to their children, we cannot make a determination about the applicability of the selfish life-cycle model unless we know the reasons for which individuals leave bequests and other intergenerational transfers to their children. Thus, in this section, we present evidence on the motives for which individuals leave bequests and other intergenerational transfers to their children in Japan and three other countries and consider whether or not the findings are consistent with the selfish life-cycle model.

If individuals are selfish, as assumed by the life-cycle model, they should either leave no bequests at all, leave only accidental or unintentional bequests arising from lifespan uncertainty, or selfish motivated bequests (see Arrondel and Masson (2006) and Laferrère and Wolff (2006)). One example of a selfishly motivated bequest is the strategic (or exchange) bequest motive proposed by Bernheim et al. (1985), whereby parents leave a bequest to their children to induce them to provide care, attention, and/or financial assistance during old age (see also Grossbard 2014). Another example of a selfishly motivated bequest is the implicit intra-family annuity contract proposed by Kotlikoff and Spivak (1981) whereby parents agree to leave a bequest to their children in return for receiving a monthly stipend from them until they die. By contrast, altruistic parents will leave a bequest to their children unconditionally (i.e., whether or not their children provide anything in return). Thus, we can shed light on the applicability of the selfish life-cycle model by looking at data on bequest motives.

The “Preference Parameters Study” of Osaka University that we referred to in the previous section collects information on bequest motives in four countries (China, India, Japan, and the United States). The survey asks “How do you feel about leaving an inheritance to your children?” and respondents are asked to select one of eight options. Two of the eight options (“I plan to leave an inheritance to my child(ren) no matter what” and “I do not plan to leave an inheritance to my child(ren) under any circumstances because doing so may reduce their will to work”) are altruistic, while four (“I plan to leave an inheritance to my child(ren) only if they provide care (including nursing care) during old age,” “I plan to leave an inheritance to my child(ren) only if they provide financial assistance during old age,” “I do not plan to make special efforts to leave an inheritance to my child(ren) but will leave whatever is left over,” and “I do not plan to leave an inheritance to my child(ren) under any circumstances because I want to use my wealth myself”) are selfish.

Horioka (2014a, 2014b) presents data on bequest motives from this survey for China, India, Japan, and the United States, and as can be seen from these papers, the proportion of respondents with an altruistic bequest motive is highest in India (75.80%) and also very high in the United States (66.97%), whereas this proportion is lowest in Japan (33.98%) and also relatively low in China (37.40%). By contrast, the proportion of respondents with a selfish bequest motive is highest in Japan (64.96%) and also relatively high in China (55.10%), whereas this proportion of lowest in India (21.82%) and also relatively low in the United States (32.76%). Thus, judging from the evidence on bequest motives, the Japanese are the most selfish among the four peoples and the Chinese are the next most selfish, whereas Indians are the most altruistic and Americans are the next most altruistic.

Moreover, as the data presented in Horioka (2014a) show, data on bequest division point to the same conclusion (i.e., that the Japanese are the most selfish and the Chinese the second most selfish). However, in the case of bequest division, Americans and Indians change positions, with Americans being the most altruistic and Indians being the second most altruistic.

Horioka et al. (1998), Horioka et al. (2000), Horioka (2002a, 2002b), Horioka (2008b, 2008c), and Horioka (2009) present similar data on bequest motives and bequest division from other surveys and obtain broadly consistent results.

Finally, Alma’amun (2012) analyzes the bequest motives of Malaysian Muslims and finds that the bequest motives of Malaysian Muslims are largely altruistic (where the responses “To make them equally well off” and “To help them regardless of their economic status” are classified as altruistic), whereas dynastic and selfish (strategic) bequest motives are of roughly equal importance.^7^

Thus, not only do the Japanese leave fewer bequests than other peoples but their bequests are more selfishly motivated that those of other peoples. These findings suggest that the selfish life-cycle model applies to a much greater extent in Japan than it does in other countries.

## Evidence from tests of altruism

The simplest version of the selfish life-cycle model assumes that individuals are selfish, not altruistic, so we can shed light on the applicability of this model by conducting tests of altruism. Thus, in this section, we survey papers that have conducted tests of altruism for the case of Japan and consider whether or not their findings are consistent with the selfish life-cycle model.

First, we survey the literature that examines the impact of parental bequest motives on the caregiving behavior of children. If children are altruistic, they should provide care, attention, and financial assistance to their parents regardless of whether or not they expect to receive bequests from them. Conversely, if children are selfish, they should provide care, attention, and financial assistance to their parents only if they expect to receive bequests from them (Bernheim et al. (1985) strategic bequest motive).

All such studies for Japan of which I am aware are consistent with the selfish life-cycle model rather than with the altruism model. For example,

1. Ohtake and Horioka (1994) find that the housing assets of parents have a positive and statistically significant impact on the probability that their children live with them and that the financial net worth of parents has a positive and statistically significant impact on the amount of the financial assistance children provide to their parents.
2. Komamura (1994) finds that the housing assets of parents have a positive and statistically significant impact on the probability that their children live with them.
3. Yamada (2006) finds that whether or not children expect to inherit a house from their parents has a positive and statistically significant impact on the probability that their children live with them and on the frequency with which their children contact them and a negative and statistically significant impact on the distance between their own home and their children’s home.
4. Wakabayashi and Horioka (2009) find that whether or not parents are managers or homeowners has a positive and statistically significant impact on the probability that their children live with them.
5. Kohara and Ohtake (2011) find that the educational attainment of parents has a positive and statistically significant impact on the probability that their children take care of them during old age.
6. Horioka et al. (2018) find that whether or not children expect to receive a bequest from their parents has a positive and statistically significant impact on the probability that they live with or near their parents and on the probability that they help their parents with housework.

If parental assets, homeownership, occupation, and educational attainment are regarded as proxies for expected bequests from parents, all of the aforementioned findings suggest that the probability of receiving bequests from parents and/or the expected amount of such bequests have a positive and statistically significant impact on the probability of parents living with, or near, their children and on the probability of receiving care, attention, and/or financial assistance from their children. This, in turn, suggests that the Japanese are selfish, not altruistic.

The seminal study on the impact of bequests on the amount of care and attention from one’s children is Bernheim et al. (1985), which finds, using data on American households from the Longitudinal Retirement History Survey (LRHS), that bequeathable wealth has a positive and significant impact on the frequency of phone calls and visits from one’s children in the case of families with two or more children, even after controlling for the parents’ age, health, and employment status, but that it has a negative and insignificant impact on the frequency of phone calls and visits in the case of families with only one child and that non-bequeathable wealth does not have a significant impact on the frequency of phone calls and visits in either sample. All of these results appear to support the selfish life-cycle model because only bequeathable wealth should influence the behavior of children and because parents’ threat of disinheritance is not credible if they have only one child.

However, Perozek (1998) replicates Bernheim et al. (1985) test using a richer data set [the 1987 National Survey of Families and Households (NSFH)] and finds that bequeathable wealth no longer has a significant impact on attention from one’s children when additional child and family characteristics are taken into account and/ or a more comprehensive measure of attention is used.

Laferrère and Wolff (2006) survey the literature from throughout the world on the impact of parental bequests on children’s caregiving behavior. They find that such research is the most prevalent in the United States and that two-thirds of such studies for the United States suggest that Americans are altruistic (see also Horioka (2014a)). Thus, our findings in this section are consistent with our earlier conclusion in Section 7 that the Japanese are more selfish than Americans.

As for studies for countries other than the United States, Laferrère and Wolff (2006) also survey studies for France, Germany, and Italy and finds that most studies for France reject altruism while two out of the three studies for Germany accept altruism, and the studies for Italy are inconclusive.

Finally, there are at least three studies of bequest division behavior—one for Japan, one for China, and one for Tanzania. Hamaaki et al. (2019) analyze the determinants of bequest division behavior in Japan using micro data from a unique survey and find that parents tend to bequeath more to children who are living with the surviving parent, which is consistent with the selfish life-cycle model, but that they do not necessarily bequeath more to economically disadvantaged children, which is evidence against the altruism model.

Turning to studies for other countries, Jiang et al. (2015) analyzes the bequest division behavior of rural households in China and find that they have mixed motives, with the likelihood of leaving bequests and the amount of the bequest being lower for children providing financial support but higher for children providing instrumental (in-kind) support. The first finding is consistent with the altruism model since children who provide financial support are presumably more affluent, but the second finding is consistent with the selfish life-cycle model.

Similarly, Wineman and Liverpool-Tasie (2019) analyze the bequest division behavior of rural households in Tanzania and find strong evidence in support of the selfish life-cycle model. For example, they find that parents with greater needs leave more to female children who reside nearby because they are more willing and able to provide attention and care to them and that they leave more to children who remit money or in-kind gifts. Moreover, they find little evidence in support of the altruism model: they find that parents do not favor children who are divorced or widowed (who are presumably more needy) and that they do not disfavor children with off-farm income.

Thus, tests of altruism that are based on examining the interrelationship between parental bequest motives, bequest division, and the caregiving behavior of children indicate the presence of considerable heterogeneity across countries, and France and Tanzania seem to be two of the few countries other than Japan in which the findings are consistent with the selfish life-cycle model.

Moreover, Hayashi (1995) conducts a completely different test of altruism using micro data from the “National Survey of Family Income and Expenditure.” If parents and children are both altruistic, the two sides should pool their incomes when deciding how much to consume, and thus if one controls for the combined income of parents and children, parental income or children’s income should not have any impact on total consumption. However, Hayashi (1995) finds, even after controlling for combined income by introducing fixed effects, that parental income and children’s income have a statistically significant impact on total consumption. This suggests that the Japanese are not pure altruists.

Thus, all of the tests of altruism surveyed here suggest that Japanese individuals are selfish, not altruistic, and that the selfish life-cycle model applies in Japan to a greater extent than it does in the United States and other countries with the possible exception of France and Tanzania.

## Evidence on the importance of borrowing (liquidity) constraints

In our brief exposition of the selfish life-cycle model in Section 2, we assumed that individuals smooth their consumption over their lifetimes, but this result requires that capital markets are perfect and that borrowing (liquidity) constraints do not exist. Thus, we can shed light on the applicability of the selfish life-cycle model by looking at how important borrowing (liquidity) constraints are in the real world. In this section, we survey the literature on the importance of borrowing (liquidity) constraints in the case of Japan and consider whether or not the findings are consistent with the selfish life-cycle model (see Hayashi 1985b, for a more general survey of this literature).

Hayashi (1985a) and Watanabe et al. (2001) find that about 15 and 25% of Japanese households face borrowing (liquidity) constraints, respectively. Similarly, Kohara and Horioka (2006) find that 8 to 15% of young Japanese couples are borrowing (liquidity) constrained. Moreover, Horioka (2012a, 2012b) and Horioka and Niimi (2020) find that the ratio of household liabilities to household disposable income in Japan was the highest among the Group of Seven (G7) countries until at least 2005.

Thus, all of the existing evidence suggests that most Japanese households are not borrowing (liquidity) constrained, that borrowing constraints do not constrain them from smoothing consumption over their lifetimes, and that the selfish life-cycle model applies in the case of Japan.^8^ This conclusion is surprising at first glance because it is well known that, during the high-growth era from the mid-1950s until the early 1970s, the Japanese government funneled capital from households to firms to enable them to finance massive amounts of investment in plant and equipment and that consumer credit was largely unavailable. It must be borne in mind, however, that after economic growth slowed, loans to households for housing and automobile purchases and other purposes expanded rapidly (see, for example, Horioka and Niimi (2020, 2021)).

## Why is there more consistent support for the selfish life-cycle model in Japan?

The overall conclusion of this paper is that the selfish life-cycle model applies in all countries but that it is more applicable in Japan than it is in the United States and most other countries. This finding suggests at first glance that the Japanese are inherently (genetically) more rational, forward-looking, and selfish than other peoples, as these are the fundamental assumptions of the selfish life-cycle model, but this is not necessarily the case. In Sections 11–15, we explore possible explanations for why there is more consistent support for the selfish life-cycle model in Japan than there is in most other countries. At least eleven possible explanations come to mind, and we group them by category into government policies, institutional factors, economic factors, demographic factors, and cultural factors.

## Government policies

### Underdeveloped social insurance system

Japan had an underdeveloped social insurance system for much of the postwar system, with the public old-age pension system being expanded only in 1973 and the public long-term care insurance system being introduced only in 2000. Moreover, the public old-age pension system has been scaled back in recent years, with the pensionable age being gradually increased from 60 to 65, contribution rates being gradually increased, and benefit levels being held down. Moreover, further reductions in benefit levels are likely as further population aging places further strains on the finances of the public old-age pension system. This means that a larger share of living expenses during retirement needs to be financed by one’s own assets in Japan, which in turn increases the need to save for life-cycle purposes (i.e. for living expenses during retirement). Thus, it could be that Japan’s underdeveloped social insurance system is one explanation for the greater importance of saving for retirement in Japan.

### High inheritance taxes

Estate, bequest, or inheritance taxes are much higher in Japan than in other countries. For example, the maximum tax rate of the inheritance tax was already high (50%) but it was increased further to 55% in 2015, and there is also a comparable gift tax that is levied on inter vivos transfers. By contrast, there is no inheritance tax at all in China and the threshold for taxable bequests is very high in the United States, as a result of which only the very wealthy are required to pay inheritance taxes in the United States. Thus, it could be that higher inheritance taxes are one explanation for why bequests are less prevalent in Japan than in other countries.

### Relatively early retirement age

Japan has traditionally had a surprising early retirement age, although it has been gradually extended over time. During most of the postwar period, the compulsory retirement age in Japan was only 55, which is much earlier than other developed countries. In 1986, the Japanese government amended the Act on Stabilization of Employment of Elderly Persons to provide various incentives for companies to raise their compulsory retirement age to 60. In 1994, the Act was further amended to legally prohibit companies from setting a compulsory retirement age below 60 beginning in 1998, and to require companies to strive to secure employment opportunities for their employees until the age of 65 beginning in 2000. In 2004, the Act was further amended to require companies with a compulsory retirement age of less than 65 to gradually provide employment opportunities for all interested employees until the age of 65 beginning in 2006, and in 2012, the Act was further amended to require companies to provide employment opportunities for all interested employees until the age of 65 beginning in 2013, either by (a) raising the compulsory retirement age, (b) introducing a continued employment system whereby employees are re-hired on a contractual basis, or (c) abolishing their compulsory retirement age entirely. Note, moreover, that even though Japan’s compulsory retirement age has been raised over time, a compulsory retirement age of 65 has not yet been fully implemented, and even a compulsory retirement age of 65 is early by international standards (for example, the United States has completely abolished compulsory retirement for almost all occupations). A relatively early retirement age implies a relatively long retirement span, *ceteris paribus*, and our theoretical analysis in Section 2 showed that a longer retirement span necessitates more life-cycle saving (saving for retirement). Thus, it could be that the relatively early retirement age in Japan is one explanation for the greater importance of saving for retirement in Japan.^9^

## Institutional factors

### The underdeveloped financial system

Japan has traditionally had a relatively underdeveloped financial system. For example, in Japan, lifetime annuities, long-term care insurance, and reverse mortgages are not provided by the private sector to the same extent as they are in other developed countries (see Horioka and Kanda 2010, for more details). Private annuities are widely available but many, if not most, of them provide for benefits only up to a certain age, meaning that they do not provide full insurance against longevity risk. This makes it necessary for Japanese households to self-insure against medical and long-term care expenses during retirement and against lifespan uncertainty, assuming that social insurance systems do not fully insure Japanese households against such risks, and this could be one explanation for the greater importance of life-cycle saving (saving for retirement) in Japan.

### Unavailability of nursing homes and professional care workers

Nursing homes and professional care workers were, until recently, less available in Japan, meaning that parents had little choice but to rely on their children for care and assistance during old age, and this could be one explanation for why selfishly motivated strategic bequest motives are more prevalent in Japan than elsewhere. Note, however, that this explanation no longer applies because the supply of nursing homes and professional care workers has increased substantially in recent years and because Japan introduced a public long-term care insurance program in 2000.

## Economic factors

### Rapid economic growth

As we discussed briefly in Section 2, the selfish life-cycle model predicts that the aggregate amount of life-cycle saving (saving for retirement) will be greater, the greater is the extent to which the lifetime incomes of workers exceeds the lifetime incomes of retirees because the aggregate amount of saving for retirement equals the saving for retirement of workers (which is presumably a function of their lifetime incomes) minus the dissaving for retirement of retirees (which is also presumably a function of their lifetime incomes). Thus, it could be that the rapid growth of household incomes during the high-growth era from the mid-1950s until the early 1970s is one explanation for the greater importance of saving for retirement in Japan during this period.

Similarly, we would expect bequests to be smaller, the greater is the extent to which the lifetime incomes of the children’s generation exceeds the lifetime incomes of the parents’ generation because the faster the growth of household incomes, the less willing even altruistic parents will be to leave bequests to their children, who are more affluent than they are. Thus, it could be that the rapid growth of household incomes during the high-growth era from the mid-1950s until the early 1970s is also one explanation for the lower prevalence of bequests in Japan during this period.

However, neither of these explanations applies any longer since economic growth in Japan has been stagnant for the past two decades or more.

## Demographic factors

### The young age structure of the population

As we showed in Section 2, the selfish life-cycle model predicts that the aggregate amount of saving for retirement will be greater, the higher is the ratio of the retirement-age population to the working-age population because workers are saving for retirement whereas retirees are dissaving for the same purpose. Moreover, as we pointed out in subsection 3.1, the ratio of the retirement-age population to the working-age population in Japan was one of the lowest among the developed countries until the mid-1980s. Thus, it could be that the unique age structure of Japan’s population is one explanation for the greater importance of life-cycle saving (saving for retirement), at least until the mid-1980s. However, this explanation no longer applies since Japan is now virtually the most aged society in the world.

### Long life expectancy

Life expectancy at birth in Japan has in recent years been virtually the longest in the world. For example, in 2019, it was 87.45 for females and 81.41 for males. The life expectancy of females was second only to Hong Kong, and that of males was the third highest in the world after Hong Kong and Switzerland (*Asahi Shimbun*, July 31, 2020). Thus, even assuming a retirement age of 65, the expected retirement span of Japanese females is 22.45 years and that of Japanese males is 16.41 years, and moreover, we need to bear in mind that the compulsory retirement age was only 60 until recently, meaning that the expected retirement span was even longer until recently. Our theoretical analysis in Section 2 showed that a longer retirement span necessitates more life-cycle saving (saving for retirement), and thus it could be that the relatively long life expectancy at birth in Japan, which entails a longer retirement span, is one explanation for the greater importance of saving for retirement in Japan.

## Cultural factors

### Weak religiosity

As the data presented in Horioka (2014a) show, the Japanese are much less religious than Americans and Indians. If weaker religiosity makes people less altruistic, it may at least partly explain the lower prevalence of altruistically motivated bequests in Japan than in the United States and India. There is a vast literature on the impact of religiosity and religious affiliation on people’s economic attitudes and outcomes starting with Max Weber’s 1905 treatise on the Protestant ethic (Weber 1905/2002) (see Guiso et al. (2003), and McCleary and Barro (2006) for comprehensive surveys of this literature). For example, Barro and McCleary (2002) find that the extent of religious beliefs has a positive impact on economic growth. Similarly, Guiso et al. (2003) conduct a careful econometric analysis of the impact of religiosity and religious affiliation on economic attitudes using data from the World Values Survey for a large number of countries and find that religious respondents differ significantly in their economic attitudes from non-religious people (for example, they place greater emphasis on thrift), even after controlling for individual country effects.

### Social norms concerning parental care

Perhaps reflecting Confucian teachings concerning the importance of filial piety, the social norm in Japan has traditionally been for children (especially sons) to take care of their elderly parents, and this could be a partial explanation for why selfishly motivated strategic bequest motives are more prevalent in Japan than in other countries.^10^ By contrast, in countries where this social norm is not as strong (for example, the United States), children will be less willing to take care of their elderly parents and hence there will be less scope for parents to induce their children to provide care by dangling a potential bequest in front of them.^11^

### Social norms concerning children’s education and marriage expenses

The social norm in Japan is for parents to pay the bulk of their children’s education and marriage expenses, and this could be one explanation for why Japanese parents are not able to leave as large a bequest to their children as parents in other countries.^12^

## Summary

In Sections 11–15, we identified a large number of not only cultural factors but also government policies, institutional factors, economic factors, and demographic factors that are capable of explaining why there is more consistent support for the selfish life-cycle model in Japan than there is in other countries. Thus, the fact that there is more consistent support for the selfish life-cycle model in Japan does not necessarily imply that the Japanese are inherently (genetically) more rational, forward-looking, and selfish than other peoples. Rather, it could be that there is more consistent support for the selfish life-cycle model in Japan because of various differences in government policies, institutional factors, economic factors, and demographic factors. Thus, it is not necessarily surprising that the selfish life-cycle model applies with greater force in a non-Western country like Japan with very different traditions, social norms, and culture and closer family ties, and this finding can potentially be explained without relying on cultural or even genetic explanations (we will say more about this in the concluding section). However, an assessment of the relative importance of the various explanations for why there is more consistent support for the selfish life-cycle model in Japan than there is in most other countries remains as a topic for further research.

## Conclusion and policy implications

In this paper, we first provided a brief exposition of the simplest version of the selfish life cycle model or hypothesis, which, together with the dynasty or altruism model, is undoubtedly the most widely used theoretical model of household behavior in economics. We then surveyed the literature on household saving behavior in Japan (with emphasis on the author’s own past research) to shed light on whether or not the selfish life-cycle model applies in the case of Japan. In particular, we surveyed the literature on a wide variety of approaches including not only studies of saving behavior (e.g., studies of the impact of the age structure of the population on the saving rate, the saving behavior of the retired elderly, saving motives, and the importance of borrowing (liquidity) constraints) but also studies of bequest behavior (e.g., studies of the prevalence of bequests, bequest motives, and tests of altruism). Almost all of the available evidence suggested that the selfish life-cycle model applies to at least some extent in all countries but that there is more consistent support for this model in Japan than there is in the United States and other countries. Thus, the answer to the question posed in the title of this paper is an unqualified “yes.”

Needless to say, selfish behavior and altruistic behavior coexist in all countries, and Japan is no exception. Fumio Hayashi, the foremost authority on Japanese saving behavior, concludes his survey paper on this topic as follows (Hayashi 1997a, p. 322):

“We can now profitably contemplate what sort of model is best suited for explaining these stylized facts [about Japanese household saving behavior]. It has become clear that intergenerational linkages through the exchange of nonmarket services and through altruism will be an essential ingredient.”

Moreover, Hayashi makes the following assertion elsewhere in the same paper (Hayashi 1997a, p. 319): “[b]oth the exchange and altruistic motives are important for explaining [Japanese household saving behavior].”

The current author agrees completely with Hayashi but strongly feels that selfish behavior is more prevalent in Japan than altruistic behavior and that there is more consistent support for the selfish life-cycle model in Japan than there is in the United States and other countries.

This paper then explored possible explanations for why there is more consistent support for the selfish life-cycle model in Japan than there is in other countries, attributing this finding to government policies, institutional factors, economic factors, demographic factors, and cultural factors.

Some readers may be surprised to learn that there is more consistent support for the selfish life-cycle model in Japan than there is in the United States and other countries because Japan is a non-Western country with very different traditions, social norms, and institutions and closer family ties. However, it must be borne in mind that the fact that family ties are close does not necessarily imply that family members harbor feelings of altruism toward one another. It could be that family ties are closer in Japan than in most other countries but that they are motivated by strategic rather than altruistic considerations (see Horioka (2019) for a more extensive discussion of the impact of culture on the saving and bequest behavior of the Japanese). Moreover, there are a large number of other differences between Japan and other countries with respect to government policies, institutional factors, economic factors, and demographic factors that are capable of explaining the greater applicability of the selfish life-cycle model in the case of Japan without having to resort to cultural or even genetic explanations.

Note, moreover, that even if social norms were a partial explanation for the greater applicability of the selfish life-cycle model in the case of Japan, it should be noted that social norms are not immutable and that they are shaped by the policy, institutional, economic, and demographic environment of the country. For example, the social norm of children taking care of their elderly parents may have arisen partly because of the unavailability of nursing homes and professional care workers and the absence of a public long-term care insurance program and it may now be weakening partly in response to the increased supply of nursing homes and professional care workers and the introduction of a public long-term care program in 2000 (see Horioka (2019) for a more detailed discussion of this argument).

Finally, we would like to consider the implications of our finding that the selfish life-cycle model applies in the case of Japan. This conclusion has at least five important implications for economic modeling and for government tax and expenditure policies:

1. When constructing theoretical models to describe Japanese household behavior, one should construct models that assume that households are selfish rather than altruistic or models that assume that selfish households and altruistic households coexist.
2. As population aging progresses even further in Japan, Japan’s household saving rate can be expected to decline ever further. This is likely to lead to substantial capital shortages, which in turn may necessitate fiscal reconstruction measures designed to reduce the large deficits (negative saving) of the government sector and may also lead to Japan running current account deficits rather than current account surpluses.
3. Our finding implies that Ricardian equivalence does not apply in the case of Japan, meaning that the Japanese Government may be able to stimulate the economy by implementing tax cuts that are financed by the issuance of government bonds (Barro 1974), but it is, of course, possible that such policies would not be effective even if Ricardian equivalence does not apply.
4. Our finding that bequests are motivated primarily by strategic or exchange considerations in Japan implies that they are accompanied by some sort of *quid pro quo* such as care, attention, and financial assistance during old age. This, in turn, implies that, in Japan, bequests from parents to children will be largely offset by transfers in the opposite direction (from children to parents), meaning that net transfers from parents to children will not necessarily be large or even positive and that wealth disparities will not necessarily be transmitted from generation to generation (from parents to children).^13^
5. Our finding that bequests are motivated primarily by strategic or exchange considerations in Japan implies that the introduction of a public long-term care insurance system in 2000 may have led to a reduction in the prevalence of bequests because parents no longer need to rely on their children for care during old age to the same extent as in the past.

Thus, whether or not the selfish life-cycle model applies in the case of Japan is important not only from an intellectual perspective but also from a policy perspective, and thus further work on this topic is of urgent need. I am therefore planning to continue my research on this topic as I enter the retirement phase of my own life cycle.

In the case of Japan, randomized controlled trials (RCTs) have not been widely used in studies of household consumption, saving, and bequest behavior. This is a promising avenue for further research, but as Ravallion (2020) has pointed out, this methodology is not necessarily the best methodology in all cases, and it should be used only if it is the best methodology for the question at hand. As Ravallion writes, “The gold standard is the best method for the question at hand.”

Given that the Covid-19 pandemic is the most pressing issue currently facing the world economy, I would like to close this article by speculating about how the pandemic will alter the extent to which the selfish life-cycle model applies in the case of Japan and other countries. First, the pandemic and the policies implemented to counteract the pandemic have caused many people to be laid off, to lose their jobs entirely, and/or to suffer sharp drops in their income. This has forced many of them to finance their living expenses by drawing down their previously accumulated wealth and will reduce the amount of wealth that they can accumulate for their retirement years, but it will also reduce the amount of wealth they can leave to their children in the form of inter vivos transfers and bequests.

Second, until the pandemic subsides, children will presumably become less likely to live with, and/or to provide care to, their parents in order to avoid infecting them with the virus, and this may cause the selfish strategic bequest motive and thence the selfish life-cycle model to become less applicable.

Thus, it is clear that the pandemic will have a pervasive impact on people’s economic behavior, but it is not clear whether it will cause the selfish life-cycle model to become more or less applicable, on balance.

## Footnotes

1 See Guiso et al. (2006) for a more general discussion of how culture affects economic outcomes by affecting people’s beliefs, values, or preferences and for a brief discussion of the impact of religiosity (an important component of culture) on attitudes toward thrift and actual saving behavior.

2 Horioka and Niimi (2017) and Niimi and Horioka (2019) point out that the rate of wealth decumulation of the retired elderly in Japan is slower than one might expect but show that this can be explained by the presence of precautionary saving and bequest motives, especially the former. See Ventura and Horioka (2020) for a similar analysis of the determinants of the saving behavior of the retired elderly in Italy.

3 The only exception to this rule is in the case of housing purchase. A household purchasing housing will decumulate financial assets in order to pay for the down payment and will incur new borrowings (a mortgage) to pay for the remainder of the cost of the house, but dissaving in these two forms will be exactly offset by saving in the form of the accumulation of real assets (viz., housing). Thus, no dissaving will occur at the time of housing purchase, but dissaving will occur after the purchase of the house in the form of depreciation.

4 It should be noted that saving for the various motives are often interrelated. For example, as noted by Wei and Zhang (2011), Du and Wei (2013), and Horioka and Terada-Hagiwara (2017), the marriage and housing motives are closely related in China and the Republic of Korea because it is common practice in these countries for parents with sons to buy housing for their sons in preparation for their marriage.

5 The second most important saving motive is the precautionary motive in both countries. Readers may be confused about why the share of net saving for housing purchase is negative in Japan. The reason is that dissaving for housing purchase is greater in absolute value than gross saving for housing purchase in Japan and the reason for this, in turn, is that housing is less durable in Japan due to differences in construction materials and thus that the depreciation of housing is greater in Japan.

6 Note, however, that saving for one’s children’s education and marriage expenses also involve intergenerational transfers and that the share of net saving for these two motives is much higher in Japan than in the United States (16.08–16.45% vs. 2.73%). Thus, the share of net saving involving intergenerational transfers is also much higher in Japan than in the United States (17.58–19.68% vs. 7.77%).

7 Alma’amun (2012) analyzes the motives for leaving bequests to family members who are excluded by Faraid (Islamic law) because the distribution of bequests among legal heirs is specified in detail in the Quran and Muslims have no discretion regarding this matter.

8 However, Ogawa (2007) finds that the proportion of households facing borrowing (liquidity) constraints has increased sharply since the mid-1990s.

9 Note, however, that even though the compulsory retirement age in Japan is relatively early, many Japanese workers continue working after formal retirement (usually at greatly reduced wages) and that self-employed workers are not bound by the compulsory retirement age.

10 Grossbard (2018) suggests that social norms regarding parental care may be influenced by the prevalence of dowries and brideprice.

11 See Banerjee et al. (2014) for an interesting analysis of how the social norm of children (especially sons) taking care of their parents has affected China’s household saving rate.

12 Note, however, that parental assistance with children’s education and marriage expenses is a form of intergenerational transfer and that, strictly speaking, it should be included in the share of bequests broadly defined in total household wealth (see footnote 5).

13 However, Niimi and Horioka (2018) find that individuals receiving bequests are more likely to leave bequests, which suggests that there is a danger than wealth disparities will be passed on from parents to children.

## References

[CR1] Alma’amun S (2012). Searching for bequest motives and attitudes to leaving a bequest among Malaysian Muslims. Jurnal Ekonomi Malaysia.

[CR2] Ando A, Modigliani F (1963). The life-cycle hypothesis of saving: aggregate implications and tests. American Economic Review.

[CR3] Arrondel, L., & Masson, A. (2006). Altruism, exchange or indirect reciprocity: what do the data on family transfers show? In S.-C. Kolm & J. M. Ythier (Eds.), *Handbook of the economics of giving, altruism and reciprocity* (pp. 971–1053). Amsterdam: Elsevier Science.

[CR4] Attanasio, O. P. (1999). Consumption. In J. B. Taylor & M. Woodford (Eds.), *Handbook of macroeconomics*, vol. 1, part B (pp. 741–812). New York, NY: Elsevier.

[CR5] Attanasio OP, Weber G (2010). Consumption and saving: models of intertemporal allocation and their implications for public policy. Journal of Economic Literature.

[CR6] Banerjee A, Meng X, Porzio T, Qian N (2014). Aggregate fertility and household savings: a general equilibrium analysis using micro data. NBER Working Paper 20050.

[CR7] Baranzini M (2005). Modigliani’s life-cycle theory of savings fifty years later. Banca Nazionale del Lavoro (BNL) Quarterly Review.

[CR8] Barro RJ (1974). Are government bonds net wealth?. Journal of Political Economy.

[CR9] Barro RJ, McCleary RM (2002). Religion and political economy in an international panel. NBER Working Paper 8931.

[CR10] Barthold, T. A., & Ito, T. (1992) Taxes and accumulation of household wealth: U.S.-Japan comparison. In T. Ito & A. Krueger (Eds.) *The political economy of tax reform* (pp. 235–290). Chicago, IL: University of Chicago Press.

[CR11] Bernheim BD, Shleifer A, Summers LH (1985). The strategic bequest motive. Journal of Political Economy.

[CR12] Birkeland, F. B. (2013). *The saving motives of Dutch households: and the effect of individual characteristics on the importance of saving motives*. Master’s thesis. Kristiansand and Grimstad: Department of Economics, Faculty of Economics and Social Sciences, University of Agder.

[CR13] Browning M, Crossley TF (2001). The life-cycle model of consumption and saving. Journal of Economic Perspectives.

[CR14] Browning M, Lusardi A (1996). Household saving: micro theories and micro facts. Journal of Economic Literature.

[CR15] Campbell D (1997). Transfer and life-cycle wealth in Japan, 1974-1984. Japanese Economic Review.

[CR16] Chao C-C, Laffargue J-P, Yu E (2011). The Chinese saving puzzle and the life-cycle hypothesis: a revaluation. China Economic Review.

[CR17] Davies JB (1981). Uncertain lifetime, consumption, and dissaving in retirement. Journal of Political Economy.

[CR18] Davies, J. B., & Shorrocks, A. F. (2000). The distribution of wealth. In A. B. Atkinson, F. Bourguignon (Eds.). *Handbook of income distribution*, vol. 1 (pp. 605–675). Amsterdam: Elsevier Science B. V.

[CR19] Deaton A (1992). Understanding consumption..

[CR20] Deaton A (2005). Franco Modigliani and the life cycle theory of consumption. Banca Nazionale del Lavoro (BNL) Quarterly Review.

[CR21] Dekle R (1989). The unimportance of intergenerational transfers in Japan. Japan and the World Economy.

[CR22] De Nardi M, French E, Jones JB (2016). Savings after retirement: a survey. Annual Review of Economics.

[CR23] Du Q, Wei S-J (2013). A theory of the competitive saving motive. Journal of International Economics.

[CR24] Feldstein M (1974). Social security, induced retirement, and aggregate capital accumulation. Journal of Political Economy.

[CR25] Garon S (1997). Modeling Japanese minds: the state in everyday life.

[CR26] Gourinchas P-O, Parker JA (2002). Consumption over the life cycle. Econometrica.

[CR27] Grossbard S (2014). A note on altruism and caregiving in the family: do prices matter?. Review of Economics of the Household.

[CR28] Grossbard S (2015). The marriage motive: a price theory of marriage: how marriage markets affect employment, consumption, and savings.

[CR29] Grossbard S (2018). An extended household model of eldercare by children and children-in-law based on Far-Eastern traditions. Review of Development Economics.

[CR30] Guiso L, Sapienza P, Zingales L (2003). People’s opium: religion and economic attitudes. Journal of Monetary Economics.

[CR31] Guiso L, Sapienza P, Zingales L (2006). Does culture affect economic outcomes?. Journal of Economic Perspectives.

[CR32] Hamaaki J, Hori M, Murata K (2019). The intra-family division of bequests and bequest motives: empirical evidence from a survey of Japanese households. Journal of Population Economics.

[CR33] Hayashi F (1985). The permanent income hypothesis and consumption durability: analysis based on Japanese panel data. Quarterly Journal of Economics.

[CR34] Hayashi F (1985). Tests for liquidity constraints: a critical survey. NBER Working Paper 1720.

[CR35] Hayashi, F. (1986). Why is Japan’s saving rate so apparently high? In S. Fischer (Ed.), *NBER Macroeconomics Annual 1986*, vol. 1 (pp. 147–210). Cambridge, MA: MIT Press.

[CR36] Hayashi F (1992). Explaining Japan’s saving: a review of recent literature. Monetary and Economic Studies.

[CR37] Hayashi F (1995). Is the Japanese extended family altruistically linked? A test based on Engel curves. Journal of Political Economy.

[CR38] Hayashi, F. (1997). Introduction to part III: a review of recent literature on Japanese saving. In F. Hayashi (Ed.), *Understanding saving: evidence from the United States and Japan* (pp. 289–329). Cambridge, MA: MIT Press.

[CR39] Hayashi F (1997). Understanding saving.

[CR40] Hayashi F, Ando A, Ferris R (1988). Life cycle and bequest savings: a study of Japanese and U.S. households based on data from the 1984 NSFIE and the 1983 Survey of Consumer Finances. Journal of Japanese and International Economies.

[CR41] Horioka CY (1984). The applicability of the life-cycle hypothesis of saving to Japan. Kyoto University Economic Review.

[CR42] Horioka CY (1985). The importance of saving for education in Japan. Kyoto University Economic Review.

[CR43] Horioka CY (1986). Why Is Japan’s private savings rate so high?. Finance and Development.

[CR44] Horioka CY (1987). The cost of marriages and marriage-related saving in Japan. Kyoto University Economic Review.

[CR45] Horioka CY (1988). Saving for housing purchase in Japan. Journal of the Japanese and International Economies.

[CR46] Horioka, C. Y. (1989). Why Is Japan’s private saving rate so high? In R. Sato & T. Negishi (Eds.), *Developments in Japanese economics* (pp. 145–178). Tokyo: Academic Press/Harcourt Brace Jovanovich.

[CR47] Horioka CY (1990). Why is Japan’s household saving rate so high? A literature survey. Journal of the Japanese and International Economies.

[CR48] Horioka CY (1991). The determinants of Japan’s saving rate: the impact of the age structure of the population and other factors. Economic Studies Quarterly (now called Japanese Economic Review).

[CR49] Horioka CY (1992). Future trends in Japan’s saving rate and the implications thereof for Japan’s external imbalance. Japan and the World Economy.

[CR50] Horioka CY, Heertje A (1993). Saving in Japan. World savings: an international survey.

[CR51] Horioka CY (1997). A cointegration analysis of the impact of the age structure of the population on the household saving rate in Japan. Review of Economics and Statistics.

[CR52] Horioka CY (2002). Are the Japanese selfish, altruistic, or dynastic?. Japanese Economic Review.

[CR53] Horioka, C. Y. (2002b). Nihonjin ha Rikotekika, Ritatekika, Ouchoutekika (Are the Japanese selfish, altruistic, or dynastic?). In K. Otsuka, M. Nakayama, S. Fukuda & Y. Honda (Eds.), Gendai Keizaigaku no Chouryuu (*Trends in Contemporary Economics*) (pp. 23–45). Tokyo: Toyo Keizai Shinposha.

[CR54] Horioka CY, Klein LR (2006). Do the elderly dissave in Japan?. Long-run growth and short-run stabilization: essays in memory of Albert Ando.

[CR55] Horioka CY, Coulmas F, Conrad H, Schad-Seifert A, Vogt G (2008). A survey of household saving behaviour. The demographic challenge—a handbook about Japan.

[CR56] Horioka CY (2008). Isan to Kakusa (Bequests and Inequality). Kikan Shakai Hoshou Kenkyuu.

[CR57] Horioka, C. Y. (2008c). Nihon ni okeru Isan Douki to Oyako Kankei: Nihonjin ha Rikotekika, Ritatekika, Ouchoutekika (Bequest motives and parent-child relations in Japan: are the Japanese selfish, altruistic, or dynastic?). In C. Y. Horioka and Institute for Research on Household Economics (Eds.), *Setainai Bunpai/ Sedaikan Iten no Keizai Bunseki (Economic analysis of intra-household distribution and intergenerational transfers)* (pp. 118–135). Kyoto: Minerva Shobo (in Japanese).

[CR58] Horioka CY (2009). Do bequests increase or decrease wealth inequalities?. Economics Letters.

[CR59] Horioka CY (2010). The (Dis)saving behavior of the aged in Japan. Japan and the World Economy.

[CR60] Horioka CY (2012). Are Japanese households financially healthy, if so, why?. Japanese Economy.

[CR61] Horioka, C. Y. (2012b). Japan and the western model: an economist’s view of cultures of household finance. In J. Logemann (Ed.), *The development of consumer credit in global perspective: business, regulation, and culture* (pp. 243–256). London: Palgrave Macmillan.

[CR62] Horioka CY (2014). Are Americans and Indians more altruistic than the Japanese and Chinese? Evidence from a new international survey of bequest plans. Review of Economics of the Household.

[CR63] Horioka CY (2014). Naze Hitobito ha Isan wo Nokosunoka? Aijou kara nanoka, Rikoshin kara nanoka? Isan Douki no Kokusai Hikaku (Who do people leave bequests? Is it out of love or selfishness? An international comparison of bequest motives). Higashi Ajia no Shiten.

[CR64] Horioka CY (2019). Are the Japanese unique? Evidence from household saving and bequest behavior. Singapore Economic Review.

[CR65] Horioka, C. Y. (2020). Nihon de Raifu Saikuru Kasetsu ha Naritatteiruka? (Is the Life Cycle Hypothesis Applicable in Japan?) In T. Ui, T. Kano, J. Doi, & T. Watanabe (Eds.). *Gendai Keizaigaku no Choryuu (Trends in Contemporary Economics)*. Tokyo: Toyo Keizai Shinposha (in Japanese).

[CR66] Horioka, C. Y., Fujisaki, H., Watanabe, W., & Ishibashi, S. (1998). Chochiku Douki/Isan Douki no Nichibei Hikaku (A U.S.-Japan Comparison of Saving Motives and Bequest Motives). In C. Y. Horioka & K. Hamada (Eds.), *Nichibei Kakei no Chochiku Koudou (A U.S.-Japan comparison of saving behavior)* (pp. 71–111). Tokyo: Nihon Hyoronsha (in Japanese).

[CR67] Horioka CY, Fujisaki H, Watanabe W, Kouno T (2000). Are Americans more altruistic than the Japanese? A U.S.-Japan comparison of saving and bequest motives. International Economic Journal.

[CR68] Horioka CY, Gahramanov E, Hayat A, Tang X (2018). Why do children take care of their elderly parents? Are the Japanese any different?. International Economic Review.

[CR69] Horioka CY, Kanda R (2010). Revitalizing the Japanese economy by socializing risk. The Japanese Economy.

[CR70] Horioka CY, Kasuga N, Yamazaki K, Watanabe W (1996). Do the aged dissave in Japan? Evidence from micro data. Journal of the Japanese and International Economies.

[CR71] Horioka CY, Niimi Y (2017). Nihon no Koureisha Setai no Chochiku Koudou ni kansuru Jisshou Bunseki (An empirical analysis of the saving behavior of elderly households in Japan). Keizai Bunseki.

[CR72] Horioka, C. Y., & Niimi, Y. (2020). Was the expansion of housing credit in Japan good or bad? *Japan and the World Economy*, 53.

[CR73] Horioka CY, Niimi Y, Mitchell OS, Lusardi AL (2021). Household debt and aging in Japan. Remaking retirement: debt in an aging economy.

[CR74] Horioka CY, Okui M (1999). A U.S.-Japan comparison of the importance and determinants of retirement saving. Economics Letters.

[CR75] Horioka CY, Terada-Hagiwara A (2012). The determinants and long-term projections of saving rates in developing asia. Japan and the World Economy.

[CR76] Horioka CY, Terada-Hagiwara A (2017). The impact of sex ratios before marriage on household saving in two Asian countries: the competitive saving motive revisited. Review of Economics of the Household.

[CR77] Horioka CY, Wan J (2007). The determinants of household saving in China: a dynamic panel analysis of provincial data. Journal of Money, Credit and Banking.

[CR78] Horioka CY, Watanabe W (1997). Why do people save? A micro-analysis of motives for household saving in Japan. Economic Journal.

[CR79] Horioka, C. Y., & Watanabe, W. (1998). Nihonjin no Mokuteki-betsu Chochiku-gaku: 1994-nen no ‘Kakei ni okeru Kin’yuu Shisan Sentaku ni kansuru Chousa’ kara no Maikuro Deta wo Mochiita Suikei (Saving amounts by motive of the Japanese: estimates based on micro data from the 1994 ‘Survey of Financial Asset Choice of Households’). In C. Y. Horioka & K. Hamada (Eds.), *Nichibei Kakei no Chochiku Koudou (A U.S.-Japan Comparison of Saving Behavior)* (pp. 29–69). Tokyo: Nihon Hyoronsha (in Japanese).

[CR80] Jappelli T, Pistaferri L (2017). The economics of consumption: theory and evidence.

[CR81] Jiang Q, Li X, Feldman MW (2015). Bequest motives of older people in rural China: from the perspective of intergenerational support. European Journal of Aging.

[CR82] Katzner DW (1999). Western economics and the economy of Japan. Journal of Post-Keynesian Economics.

[CR83] Katzner DW (2008). Culture and economic explanation: economics in the US and Japan.

[CR84] Koga M (2006). The decline of Japan’s saving rate and demographic effects. Japanese Economic Review.

[CR85] Kohara M, Horioka CY (2006). Do borrowing constraints matter? An analysis of why the permanent income hypothesis does not apply in Japan. Japan and the World Economy.

[CR86] Kohara M, Ohtake F (2011). Altruism and the care of elderly parents. The Japanese Economy.

[CR87] Komamura K (1994). Koreisha Kakei ni okeru Isan Koudou no Keizai Bunseki (An economic analysis of bequest behavior in elderly households). Kikan Shakai Hoshou Kenkyuu.

[CR88] Kotlikoff LJ, Spivak A (1981). The family as an incomplete annuities market. Journal of Political Economy.

[CR89] Kotlikoff LJ, Summers LH (1981). The role of intergenerational transfers in aggregate capital accumulation. Journal of Political Economy.

[CR90] Laferrère, A., & Wolff, F.-C. (2006). Microeconomic models of family transfers. In S.-C. Kolm & J. M. Ythier (Eds.), *Handbook of the economics of giving, altruism and reciprocity*, vol. 2 (pp. 889–969). Amsterdam: Elsevier Science B.V.

[CR91] Loayza N, Schmidt-Hebbel K, Serven L (2000). What drives private saving across the World. Review of Economics and Statistics.

[CR92] McCleary RM, Barro RJ (2006). Religion and economy. Journal of Economic Perspectives.

[CR93] Modigliani F (1966). The life cycle hypothesis of saving, the demand for wealth and the supply of capital. Social Research.

[CR94] Modigliani, F. (1975). The life-cycle hypothesis of saving twenty years later. In M. Parkin (Ed.), *Contemporary issues in economics* (pp. 2–35). Manchester: Manchester University Press.

[CR95] Modigliani F, Brumberg RH, Kurihara KK (1954). Utility analysis and the consumption function: an interpretation of cross-section data. Post-Keynesian Economics.

[CR96] Modigliani, F., & Brumberg, R. H. (1980). Utility analysis and aggregate consumption functions: an attempt at integration. In A. Abel (Ed.), *The collected papers of Franco Modigliani, vol. 2: the life cycle hypothesis of saving* (pp. 128–197). Cambridge, MA: The MIT Press.

[CR97] Modigliani F, Cao SL (2004). The Chinese saving puzzle and the life-cycle hypothesis. Journal of Economic Literature.

[CR98] Morishima M (1982). Why has Japan succeeded? Western technology and the Japanese ethos.

[CR99] Niimi Y, Horioka CY (2018). The impact of intergenerational transfers on wealth inequality in Japan and the United States. World Economy.

[CR100] Niimi Y, Horioka CY (2019). The wealth decumulation behavior of the retired elderly in Japan: the relative importance of precautionary saving and bequest motives. Journal of the Japanese and International Economies.

[CR101] Ogawa K (2007). Why did Japan’s household savings rate fall in the 1990s. Applied Economics.

[CR102] Ogawa, K., & Horioka, C. Y. (1996). Shouhi/Chochiku (consumption and saving). In K. Kaizuka, Y. Kosai & Y. Nonaka (Eds.), Nihon Keizai Jiten (Japanese Economic Encyclopedia) (pp. 965–980). Tokyo: Nihon Keizai Shinbunsha (in Japanese).

[CR103] Ohtake, F., & Horioka, C. Y. (1994). Chochiku Douki (Saving motives). In T. Ishikawa (Ed.), Nihon no Shotoku to Tomi no Bunpai (The distribution of income and wealth in Japan) (pp. 211–244). Tokyo: Tokyo Daigaku Shuppankai (in Japanese).

[CR104] Perozek MG (1998). A rexamination of the strategic bequest motive. Journal of Political Economy.

[CR105] Ravallion M (2020). Should the randomistas (continue to) rule? NBER Working Paper 27554.

[CR106] Schunk D (2009). What determines the saving behavior of German households: an examination of saving motives and saving decisions. Journal of Economics and Statistics (Jahrbuecher fuer Nationaloekonomie und Statistik).

[CR107] Ventura L, Horioka CY (2020). The wealth decumulation behavior of the retired elderly in Italy: the importance of bequest motives and precautionary saving. Review of Economics of the Household.

[CR108] Wakabayashi M, Horioka CY (2009). Is the eldest son different? The residential choice of siblings in Japan. Japan and the World Economy.

[CR109] Watanabe K, Watanabe T, Watanabe T (2001). Tax policy and consumer spending: evidence from Japanese fiscal experiments. Journal of International Economics.

[CR110] Weber M (1905). The protestant ethic and the spirit of capitalism (translated by Peter Baehr and Gordon C. Wells).

[CR111] Wei S-J, Zhang X (2011). The competitive saving motive: evidence from rising sex ratios and savings rates in China. Journal of Political Economy.

[CR112] Weil DN (1994). The saving of the elderly in micro and macro data. Quarterly Journal of Economics.

[CR113] Wineman A, Liverpool-Tasie LS (2019). All in the family: bequest motives in rural Tanzania. Economic Development and Cultural Change.

[CR114] Yamada K (2006). Intra-family transfers in Japan: intergenerational co-residence, distance, and contact. Applied Economics.

[CR115] Yao R, Wang F, Weagley RO, Liao L (2011). Household saving motives: comparing American and Chinese consumers. Family and Consumer Sciences Research Journal.

